# A computational approach to identify transposable element insertions in cancer cells

**DOI:** 10.1186/gb-2011-12-s1-p28

**Published:** 2011-09-19

**Authors:** Israel T Silva, Daniel G Pinheiro, Wilson A Silva

**Affiliations:** 1 Regional Blood Center of Ribeirão Preto, Molecular Biology and Bioinformatics Laboratory, Ribeirão Preto, São Paulo 14051-140, Brazil; 2 Barão de Mauá University, Ribeirão Preto, São Paulo 14026-150, Brazil; 3 Department of Genetics, Medical School of Ribeirão Preto, University of São Paulo, Ribeirão Preto, São Paulo 14049-900, Brazil

## Background

Transposable elements (TEs) in the human genome may contribute to molecular evolution, hereditary diseases and cancer [1-3]. Therefore, analyzing the impact of TEs in the genome is necessary to better characterize genetic events related to tumorigenesis. Here, we used a computational approach to identify TE insertions in publicly available data for exome sequences in lymphoblastoid and breast tumor cells derived from the same patient.

## Methods

A total of 29,340, sequences from the cell lines HCC1954 (18,365,271) and HCC1954BL (10,975,107) were used to investigate gene fusion with TEs (gfTEs) [4,5]. The RepeatMasker and Burrows-Wheeler Alignment (BWA) tools were used to identify and to map gfTEs, respectively. We also used BEDTools to find overlaps between gfTEs and genome annotations. Human mRNAs and RepeatMasker tracks were downloaded in BED format from the GRCh37/ hg19 assembly. Repbase was used to filter the eukaryotic TEs.

## Results

RepeatMasker was used to identify gfTEs in the exome reads. Next, the repeat masked reads were aligned against the reference genome using BWA. Finally, we filtered the aligned reads to exclude those without TEs (length of Ns <15, Ns means block of nucleotides masked), those with alignments showing low sequence identity (<95%) or those with a small hit length (<50 nucleotides). The study focused on the detection of TEs in coding sequence gene regions. A total of 3,307,608 reads were excluded, and 23,841 reads were predicted as cancer-specific gfTEs. Table 1 shows the number of gfTEs distributed among the TE families and highlights the members with higher frequency in both cell lines. Insertions of LINE/L1 and SINE/Alu were the most frequent. The Gene Ontology analysis for the biological process and molecular function terms showed a bias toward membrane receptor and cell adhesion proteins.

**Table 1:** Number of genes containing insertion of TEs from different families

Class/Family	HCC1954BL (N)	HCC1954 (T)
DNA	4	3
DNA/MuDR	5	1
DNA/PiggyBac	2	2
DNA/TcMar-Mariner	10	9
DNA/TcMar-Tc2	6	8
DNA/TcMar-Tigger	90	96
DNA/hAT	2	8
DNA/hAT-Blackjack	7	19
DNA/hAT-Charlie	107	137
DNA/hAT-Tip100	12	19
LINE/CR1	23	25
LINE/Dong-R4	1	1
LINE/L1	863	641
LINE/L2	163	175
LINE/RTE	9	13
LINE/RTE-BovB	1	0
LTR	1	2
LTR/ERV1	134	145
LTR/ERVK	11	17
LTR/ERVL	70	77
LTR/ERVL-MaLR	148	186
LTR/Gypsy	6	7
Other	5	4
RNA	1	3
SINE	6	17
SINE/Alu	264	406
SINE/Deu	5	14
SINE/MIR	109	145
SINE/tRNA	0	3
Satellite	7	15
Satellite/acro	2	1
Satellite/centr	52	112
Unknown	6	8
rRNA	14	11
scRNA	4	2
snRNA	0	2
srpRNA	4	1
tRNA	0	1
Total	2.154	2.340

## Conclusions

We used a computational approach to identify putative cancer-specific gfTEs using human exome capture sequences. Interestingly, the total number of gfTEs was similar in normal and tumor cell lines, but the Gene Ontology analysis revealed an enrichment of insertions in genes encoding protein receptors and cell adhesion molecules. These results suggest that TEs could be contributing to cancer development.

## References

[B1] Cordaux R Batzer MA The impact of retrotransposons on human genome evolution Nat Rev Genet 2009 10 691 703 10.1038/nrg2640 19763152 PMC2884099

[B2] Callinan PA Batzer MA Retrotransposable elements and human disease Genome Dyn 2006 1 104 115 18724056 10.1159/000092503

[B3] Zhang W Edwards A Fan W Deininger P Zhang K Alu distribution and mutation types of cancer genes BMC Genomics 2011 12 157 10.1186/1471-2164-12-157 21429208 PMC3074553

[B4] Zhao Q Kirkness EF Caballero OL Galante PA Parmigiani RB Edsall L Kuan S Ye Z Levy S Vasconcelos AT Ren B de Souza SJ Camargo AA Simpson AJ Strausberg RL Systematic detection of putative tumor suppressor genes through the combined use of exome and transcriptome sequencing Genome Biol 2010 11 R114 10.1186/gb-2010-11-11-r114 21108794 PMC3156953

[B5] Galante PA Parmigiani RB Zhao Q Caballero OL de Souza JE Navarro FC Gerber AL Nicolás MF Salim AC Silva AP Edsall L Devalle S Almeida LG Ye Z Kuan S Pinheiro DG Tojal I Pedigoni RG de Sousa RG Oliveira TY de Paula MG Ohno-Machado L Kirkness EF Levy S da Silva WA Jr Vasconcelos AT Ren B Zago MA Strausberg RL Simpson AJ de Souza SJ Camargo AA Distinct patterns of somatic alterations in a lymphoblastoid and a tumor genome derived from the same individual Nucleic Acids Res 2011 39 6056 6068 doi: 10.1093/nar/gkr221 10.1093/nar/gkr221 21493686 PMC3152357

